# Non-invasive ventilation in severe acute respiratory failure complicating Madelung’s disease: a case series and management review

**DOI:** 10.3389/fmed.2026.1805166

**Published:** 2026-06-04

**Authors:** Bruno Gomes, Antonio M. Esquinas, Fares N. Alwajeeh

**Affiliations:** 1 Hospital Lusíadas, Porto, Portugal; 2 Intensive Care Unit, Hospital Meseguer, Murcia, Spain; 3 Department of Respiratory Care, Alrazi University & 21 September University, Sana'a, Yemen

**Keywords:** acute respiratory failure, case report, COVID-19, difficult airway, Madelung’s disease, non-invasive ventilation, obstructive sleep apnea

## Abstract

Madelung’s disease (Launois-Bensaude syndrome) presents a critical challenge when managing acute respiratory failure (ARF) due to the high risk of difficult airway and failed intubation posed by symmetric cervical lipomatosis. This case series investigates the feasibility of non-invasive ventilation (NIV) as a primary strategy for ARF in these high-risk patients. We present two cases of severe ARF triggered by COVID-19 pneumonia in patients with Madelung’s disease. Both patients were successfully managed with NIV using a total face mask, which avoided the need for hazardous endotracheal intubation. Key challenges included managing bilateral pneumothoraxes as a complication of NIV in one patient, and identifying and treating severe obstructive sleep apnea (OSA), a prevalent comorbidity as a crucial factor for successful weaning in the other. Our findings, integrated with a review of existing literature, demonstrate that a proactive NIV-first approach is a viable and potentially life-saving strategy in this population. This report provides a practical management framework, emphasizing careful interface selection, vigilance for complications, and systematic screening for underlying OSA to guide clinicians in optimizing outcomes for these complex patients.

## Introduction

Madelung’s disease, also known as Launois-Bensaude syndrome, is a rare disorder characterized by the symmetrical, non-encapsulated accumulation of adipose tissue around the neck, shoulders, and upper trunk (1). This unique anatomy creates a critical challenge when managing acute respiratory failure (ARF). The fatty deposits can cause severe upper airway obstruction and distort oropharyngeal anatomy, classifying these patients as having a recognized “difficult airway” with a high risk of failed endotracheal intubation (2, 3).

The convergence of this high-risk condition with the COVID-19 pandemic, which frequently leads to severe hypoxemic ARF, created a complex clinical scenario for which there are no established management guidelines. Non-invasive ventilation (NIV) presents a theoretically attractive strategy to avoid hazardous intubation, but practical evidence on its application in this population is scarce (4, 5).

Madelung’s disease is an extremely rare condition, with limited published data on its management during acute respiratory failure. Furthermore, the association between Madelung’s disease and COVID-19-related respiratory failure has been scarcely described in the medical literature. This scarcity of evidence makes our case series particularly valuable for clinicians facing similar challenging airway scenarios.

This report details the clinical course of these two patients, focusing on the use of NIV as the primary ventilatory strategy. We describe the challenges encountered, including the development of pneumothorax and the unmasking of severe obstructive sleep apnea, and integrate these experiences with a review of the literature. Our aim is to provide a structured management framework for ARF in patients with Madelung’s disease, emphasizing a strategy that prioritizes the avoidance of high-risk airway interventions.

## Case presentation

### Case 1

A 65-year-old male with a known history of Madelung’s disease presented to the emergency department with acute hypoxemic respiratory failure secondary to confirmed COVID-19 pneumonia. According to the Schlitz classification, the patient’s lipomatosis corresponds to Type II (pseudoathletic type), characterized by symmetric fatty deposits around the neck, shoulders, and upper trunk without visceral involvement. Neck circumference was 52 cm, mouth opening was limited to 3 cm, and neck extension was restricted to approximately 15 degrees. Thoracic lipomatosis was not significant; therefore, chest tube placement was not technically challenging. His body mass index (BMI) was 30 kg/m^2^. Cervical and thoracic CT imaging was not performed during the acute phase due to clinical instability and the risk of patient transport.

Upon admission, his vital signs were indicative of severe distress: temperature 38.7 °C, respiratory rate 37 breaths/min, heart rate 120 bpm, blood pressure 140/60 mmHg, and an oxygen saturation (SpO₂) of 82% on room air. Arterial blood gas (ABG) analysis revealed a PaO₂ of 42 mmHg and a PaO₂/FiO₂ ratio of 140, meeting criteria for acute respiratory distress syndrome (ARDS) according to the Berlin definition (6, 7).

Given the high risk of difficult or failed intubation associated with Madelung’s disease, a strategy of NIV was initiated as first-line therapy. He was admitted to the intensive care unit (ICU) and placed on a bilevel positive airway pressure (BiPAP) device (V60, Philips Respironics) via a total face mask. Settings were titrated to an inspiratory positive airway pressure (IPAP) of 18 cm H₂O and an expiratory positive airway pressure (EPAP) of 8 cm H₂O to achieve adequate tidal volumes and maintain SpO₂ between 92 and 94%.

His ICU course was complicated by the development of bilateral pneumothoraces on day 3 of NIV therapy, which was managed conservatively with thoracic drainage. The pneumothorax was moderate in severity (approximately 30% on chest imaging) and was managed with a single 16-French chest tube placed in the right hemithorax. Following drainage, NIV settings were adjusted by reducing IPAP from 18 to 14 cm H₂O to limit peak pressures while maintaining adequate tidal volumes (6–8 mL/kg). The air leak resolved completely by day 7, and the chest tube was removed on day 9. Despite this complication, NIV was continued. The patient showed gradual improvement, allowing for de-escalation to high-flow nasal oxygen by day 7, and subsequently to conventional low-flow oxygen. He was successfully discharged from the ICU to a general pneumonia ward on day 12 (Table 1). A sleep study was not performed during hospitalization as the patient had no clinical symptoms suggestive of obstructive sleep apnea (no witnessed apneas, excessive daytime sleepiness, or snoring reported by family).

**Table 1:** Summary of case report.

Feature/parameter	Case 1	Case 2	Clinical implication/insight
Age/sex	65-year-old male	70-year-old male	
Primary trigger for ARF	Severe COVID-19 pneumonia (ARDS criteria)	Severe COVID-19 pneumonia with bacterial superinfection	Highlights a common modern precipitant of ARF in vulnerable patients
Initial ABG (PaO₂/FiO₂)	140 (Severe ARDS) (6)	286 (Moderate ARDS) (6)	Both cases presented with significant hypoxemia justifying ventilatory support.
Primary ventilatory strategy	First-Line (BiPAP via Total Face Mask) NIV	First-Line (BiPAP via Oronasal Mask)	Active avoidance of intubation due to known difficult airway. Case 1 demonstrates interface choice to prevent skin lesions.
Major complication during NIV	Bilateral pneumothorax	None	Shows NIV can be sustained even with complications; also reveals underlying OSA as a cause of prolonged failure.
Discharge outcome	Successful weaning, discharged from ICU	Discharge with home APAP after OSA diagnosis	NIV was a bridge to recovery in Case 1 and a bridge to diagnosis in Case 2
Key morbidity	Madelung’s disease, Obesity (BMI 30)	Madelung’s disease, Severe OSA (AHÍ = 26), Heavy smoking/alcohol history	Case 2 underscores the critical link between Madelung’s and undiagnosed OSA, a major factor in weaning failure.

ARF, acute respiratory failure; ARDS, acute respiratory distress syndrome; ABG, arterial blood gas; NIV, non-invasive ventilation; BiPAP, bilevel positive airway pressure; APAP, auto-adjusting positive airway pressure; OSA, obstructive sleep apnea; BMI, body mass index; AHI, apnea-hypopnea index.

### Case 2

A 70-year-old male with a long-standing history of Madelung’s disease (status post excision of multiple cervical lipomas) was admitted with dyspnea at rest and a dry cough. According to the Schlitz classification, the patient’s lipomatosis corresponds to Type II (pseudoathletic type) with cervical predominance (7). Neck circumference was 58 cm, and anatomical landmarks for intubation were completely obliterated.

CT imaging was not available at the time of admission; therefore, anatomical assessment was based on physical examination and clinical findings.

He had a significant history of heavy alcohol use (now abstinent for 6 years) and a 55 pack-year smoking history. Physical examination was notable for profound cervical dysmorphia with marked enlargement of the neck circumference. Initial SpO₂ was 88–90% on room air. He was diagnosed with severe COVID-19 pneumonia with bacterial superinfection.

Due to progressive global respiratory failure and the anticipated difficult airway, NIV was commenced using an oronasal mask (Stellar 150 ventilator, ResMed) in bilevel spontaneous-timed (ST) mode. Settings were IPAP 16 cm H₂O, EPAP 8 cm H₂O, with a backup rate. Weaning from ventilatory support proved challenging, punctuated by periods of gas exchange deterioration and significant nocturnal desaturation. The ST mode was selected because the patient exhibited an unreliable respiratory drive during sleep, with episodes of central hypopnea. This contrasts with Case 1, where spontaneous mode was sufficient as the patient maintained a consistent respiratory drive. The backup rate (set at 12 breaths/min) ensured adequate minute ventilation during these episodes, preventing nocturnal desaturation and reducing the work of breathing.

A diagnostic workup, including a cardio-respiratory sleep study performed after hospital discharge (at 4 weeks post-discharge), revealed a previously undiagnosed moderate obstructive sleep apnea (OSA) with an apnea-hypopnea index (AHI) of 26 events/h. This underlying condition was identified as a major contributor to the prolonged respiratory failure and difficult weaning.

The patient was subsequently established on home auto-adjusting positive airway pressure (APAP) therapy, which resulted in sustained clinical and gasometric improvement, facilitating his eventual discharge with outpatient follow-up (Table 1).

## Discussion

This case series demonstrates the feasibility and challenges of employing NIV as a primary strategy for managing ARF in patients with Madelung’s disease, a population at exceptionally high risk during emergency airway management.

High-flow nasal oxygen was considered for both patients but was not selected as the initial therapy for two reasons: (1) Case 2 had a hypercapnic component (global respiratory failure), and high-flow nasal oxygen does not provide ventilatory support for CO₂ clearance; (2) in both cases, the anticipated need for inspiratory pressure support to overcome increased upper airway resistance caused by cervical lipomas favored BiPAP over high-flow nasal oxygen.

Continuous positive airway pressure (CPAP) was also considered but not chosen because CPAP delivers a single fixed pressure without inspiratory assistance. In patients with Madelung’s disease, the fatty infiltration around the neck and pharynx increases upper airway resistance, requiring inspiratory pressure support to maintain adequate tidal volumes. BiPAP provides both IPAP to assist ventilation and EPAP to maintain alveolar recruitment and oxygenation.

Fonseca et al. (3) successfully used nasal pillows for CPAP in a patient with Madelung’s disease and OSA. However, in the acute setting of severe hypoxemic respiratory failure, our Case 1 required higher inspiratory pressures (IPAP 18 cm H₂O) to achieve adequate tidal volumes, which is better delivered via a total face mask. Therefore, we recommend BiPAP as first-line therapy in acute ARF, with CPAP reserved for stable OSA or as a step-down after weaning from BiPAP.

The paramount concern in managing ARF in Madelung’s disease is the well-documented high incidence of difficult or failed endotracheal intubation (1–3). The symmetric cervical lipomatosis distorts anatomy, limiting neck extension and obscuring laryngeal views. Our management decision to initiate NIV first-line in both cases was driven directly by this imperative. The successful outcome avoiding intubation entirely strengthens the argument that NIV should not be merely an alternative but a primary and deliberate strategy in the initial management plan for these patients, whenever clinically permissible.

Specific airway challenges in Madelung’s disease include limited neck extension (often <15 degrees), restricted mouth opening (<3 cm), distorted laryngeal anatomy due to fatty infiltration, and unexpected difficult mask ventilation. These factors lead to a high incidence of failed intubation.

Despite the success of NIV in our two cases, clinicians must be prepared for NIV failure. Based on the literature (8–10) and our experience, we recommend the following stepwise approach:

1. *Preparation*: Ensure a videolaryngoscope (e.g., GlideScope) is immediately available.
2. *Supraglottic airway devices*: Have a range of supraglottic devices (e.g., laryngeal mask airway, i-gel) ready as a rescue option.
3. *Awake fiberoptic intubation*: If time permits and the patient is cooperative, awake fiberoptic intubation is the safest approach.
4. *Surgical airway*: A low threshold for surgical airway (cricothyroidotomy or tracheostomy) should be maintained, with a surgical airway kit immediately available at the bedside. Preoperative marking of anatomical landmarks with ultrasound is recommended.
5. *Team briefing*: A pre-induction briefing with the entire team (anesthesia, ICU, surgery) should be conducted.

Successful NIV in this context requires careful technical execution. In Case 1, the use of a total face mask was instrumental. Patients with Madelung’s disease often have irregular facial contours and sensitive skin due to underlying lipomas. The total face mask distributes pressure over a larger area, which likely contributed to the absence of skin breakdown in our patient, a complication reported with other interfaces (3). Furthermore, clinicians must be vigilant for complications common to both NIV and the underlying condition. The development of bilateral pneumothoraces in Case 1, while serious, was managed without intubation, demonstrating that certain NIV complications can be managed conservatively without abandoning the non-invasive strategy.

In patients with Type I or Type III Madelung’s disease (7), where lipomatosis extends to the thorax, traditional chest tube placement may be technically challenging due to overlying adipose tissue obscuring anatomical landmarks. Based on the literature (8, 9) and our experience, we recommend the following stepwise approach:

1. *Ultrasound guidance*: Bedside thoracic ultrasound should be used to identify the optimal insertion site, avoiding areas with thick subcutaneous lipomas.
2. *Smaller-bore catheters*: A smaller-bore catheter (e.g., 12–14 French) inserted via a Seldinger technique may be preferred over a large-bore chest tube.
3. *Surgical consultation*: Early consultation with thoracic surgery is recommended, as some patients may require video-assisted thoracoscopic surgery (VATS).
4. *Needle decompression*: In tension pneumothorax, needle decompression should be performed using a longer catheter (e.g., 8–10 cm) to reach the pleural space through the thickened chest wall.

The most significant insight from Case 2 is the elucidation of OSA as a central comorbidity shaping the clinical course. The patient’s difficult weaning and nocturnal hypoventilation were initially puzzling until a formal sleep study revealed moderate OSA (AHI 26). This finding is not coincidental but a recognized association in Madelung’s disease, thought to be caused by fatty infiltration compromising upper airway patency (4, 5).

Our case powerfully illustrates that ARF in these patients may be an acute-on-chronic phenomenon. Therefore, the management algorithm must extend beyond treating the acute precipitant (e.g., pneumonia). We propose that routine screening for OSA, either clinically or via sleep studies upon clinical stabilization, should be integrated into the care pathway. As shown, treating the underlying OSA with home positive airway pressure therapy was transformative for the patient’s long-term respiratory status.

Based on our experience and literature synthesis (1–7), we propose structured approach to ARF in Madelung’s disease: (1) Early identification of Madelung’s disease as a “difficult airway alert” upon admission; (2) A trial of NIV with a carefully selected interface (consider total face mask) as the initial strategy for moderate to severe ARF; (3) Vigilance for complications like pneumothorax; (4) Investigation for concomitant OSA in cases of prolonged weaning; and (5) Long-term planning including management of chronic respiratory issues like OSA.

This case series provides the first structured description of a primary NIV-first strategy in patients with Madelung’s disease. The detailed documentation of NIV settings, interface selection (total face mask), and management of complications (pneumothorax) offers practical guidance for clinicians facing similar high-risk airway scenarios. The identification of underlying OSA as a key comorbidity and its impact on weaning represents a novel clinical insight that extends the literature beyond acute management to include long-term respiratory care.

Our conclusions are drawn from a limited number of cases (*n* = 2), which is inherent to studying rare diseases. Furthermore, the specific challenge was ARF triggered by COVID-19; outcomes may vary with other etiologies. Nevertheless, the principles of airway risk mitigation and comprehensive search for contributing factors like OSA remain broadly applicable. While the small sample size limits generalizability, the consistent success of the NIV-first strategy across both cases, despite different presentations, supports the robustness of the approach. The detailed case presentation with objective data (ABG, imaging, sleep study) enhances transparency and allows readers to assess the applicability to their own clinical practice.

## Patient perspective

Both patients expressed profound relief at avoiding invasive endotracheal intubation, a procedure they were informed carried exceptionally high risk due to their anatomical condition. For the patient in Case 2, the diagnosis of OSA was a pivotal moment. He reported that prior to this admission, his chronic fatigue and unrefreshing sleep were unexplained burdens. The initiation of home APAP therapy not only resolved his nocturnal symptoms but also provided a clear understanding of a long-standing health issue, significantly improving his overall quality of life and engagement with his long-term health management. These perspectives underscore that successful management in such complex cases extends beyond acute physiological support to include diagnosis of underlying comorbidities and patient education.

## Conclusion

In conclusion, managing ARF in Madelung’s disease requires a paradigm shift away from default intubation. A proactive NIV-first strategy, informed by an understanding of the difficult airway and a high index of suspicion for comorbid OSA, can successfully avert life-threatening procedures and guide more holistic patient care.

## Data Availability

The raw data supporting the conclusions of this article will be made available by the authors, without undue reservation.
